# Nascent osteoblast matrix inhibits osteogenesis of human mesenchymal stem cells *in vitro*

**DOI:** 10.1186/s13287-015-0223-x

**Published:** 2015-12-22

**Authors:** Catherine M. Kolf, Lin Song, Jeannine Helm, Rocky S. Tuan

**Affiliations:** Department of Health and Human Services, Cartilage Biology and Orthopaedics Branch, National Institute of Arthritis and Musculoskeletal and Skin Diseases, National Institutes of Health, Bethesda, MD USA; Department of Biology, Johns Hopkins University, Baltimore, MD USA; Department of Orthopaedic Surgery, Center for Cellular and Molecular Engineering, University of Pittsburgh School of Medicine, 450 Technology Drive, Room 221, Pittsburgh, PA 15219 USA; Johns Hopkins Medicine, Media Relations and Public Affairs, 901 S. Bond St., Suite 550, Baltimore, MD 21231 USA; Stryker Orthopedics, 325 Corporate Drive, Mahwah, NJ 07430 USA; Translational Genomics Research Branch, National Institute for Dental and Craniofacial Research, National Institutes of Health, Bethesda, MD 20892 USA

**Keywords:** Mesenchymal stem cells, Osteoblasts, Extracellular matrix, Osteogenesis, Stem cell niche

## Abstract

**Introduction:**

Adult mesenchymal stem cells (MSCs) are considered promising candidates for cell-based therapies. Their potential utility derives primarily from their immunomodulatory activity, multi-lineage differentiation potential, and likely progenitor cell function in wound healing and repair of connective tissues. However, *in vitro*, MSCs often senesce and spontaneously differentiate into osteoblasts after prolonged expansion, likely because of lack of regulatory microenvironmental signals. *In vivo*, osteoblasts that line the endosteal bone marrow surface are in close proximity to MSCs in the marrow stroma and thus may help to regulate MSC fate.

**Methods:**

We examined here how osteogenic differentiation of MSCs *in vitro* is affected by exposure to osteoblastic cells (OBCs). Human bone marrow MSCs were exposed to OBCs, derived by induced osteogenic differentiation of MSCs, either directly in contact co-cultures, or indirectly to OBC-conditioned medium or decellularized OBC extracellular matrix (ECM).

**Results:**

Our results showed that OBCs can act as negative regulators of MSC osteogenesis. mRNA expression profiling revealed that OBCs did not affect MSC osteogenesis in direct contact cultures or via secreted factors. However, seeding MSCs on decellularized OBC ECM significantly decreased expression of several osteogenic genes and maintained their fibroblastic morphologies. Proteomic analysis identified some of the candidate protein regulators of MSC osteogenesis.

**Conclusions:**

These findings provide the basis for future studies to elucidate the signaling mechanisms responsible for osteoblast matrix-mediated regulation of MSC osteogenesis and to better manipulate MSC fate *in vitro* to minimize their spontaneous differentiation.

**Electronic supplementary material:**

The online version of this article (doi:10.1186/s13287-015-0223-x) contains supplementary material, which is available to authorized users.

## Introduction

Mesenchymal stem cells (MSCs) are multipotent cells resident in adult tissues that function in wound healing and repair of connective tissues, especially the musculoskeletal system. Capable of extensive expansion in vitro [1], they are an exciting cell type to study because of their therapeutic potential in tissue engineering and regenerative medicine [2]. MSCs are commonly differentiated into osteoblasts, chondrocytes and adipocytes [3], as well as other cell types [4, 5]. Still more promising, MSCs have been shown to have immunomodulatory properties, allowing them to evade the immune system when transplanted [6].

A practical hurdle in the application of MSCs is their tendency to undergo spontaneous differentiation upon extended propagation in vitro. Specifically, osteogenesis appears to be the default differentiation pathway for long term cultures of MSCs [7], causing them to senesce and lose their therapeutic potential. However, in vivo, both of these processes are prevented due to the specialized niche in which MSCs reside. Recapitulation of this niche in vitro would permit more extensive expansion of MSCs without the loss of their multi-lineage differentiation potential. The feasibility of such an endeavor requires understanding the nature of the MSC niche.

In the bone marrow, the MSC niche is, at least partially, perivascular [8]. However, MSCs are likely in contact with both endothelial cells and osteoblasts (OBs), which line the bone marrow cavity. Since OBs have been implicated in the hematopoietic stem cell niche [9, 10], it seems likely that they could also be key players in the MSC niche. A number of studies have assessed the effects of OBs and their secreted soluble and extracellular matrix (ECM) proteins on MSC osteogenesis [11–15]. For example, co-culturing of embryonic mouse MSCs and OBs, separated in a Transwell set-up, showed that factors secreted by OBs can cause a 30-fold increase in MSC proliferation and a decrease in their alkaline phosphatase (ALP) activity, indicative of less osteogenic differentiation [11]. While the active factors responsible for this observed effect have not been ascertained, both bone morphogenetic proteins (BMPs) and insulin-like growth factors (IGFs) are secreted by OBs and have been implicated in several niche functions in other systems, including stem cell maintenance [16-18] and asymmetric cell division [19].

On the other hand, both BMPs and IGFs can also be found sequestered in the ECM of OBs; in fact, BMPs were first identified in demineralized bone matrix (DBM) because of their activity in inducing bone formation [20]. While several ECM molecules, in their purified forms, have been shown to enhance bone formation (e.g., collagen type I [21]), the reactivity of MSCs to native OB matrix, with its three-dimensionality and protein-protein interactions, is likely to be more complex. In addition, it is important to examine the interactions between human MSCs and *nascent* human OBs, the most likely osteoblastic cell type to interact with MSCs in the native bone marrow environment. Mature bone cells, such as fully differentiated OBs and osteocytes, from which DBM is created, are encased in bony matrix and are most likely exposed to MSCs only upon tissue injury, such as bone fracture. This information serves as the foundation for our study of MSC osteogenesis, in which we tested the effects of relatively immature osteoblastic cells (OBCs), derived in vitro from MSCs. Specifically, we have examined the influence of cell-cell interaction, and secreted factors and extracellular matrix produced by the OBCs on MSC osteogenic differentiation.

## Materials and methods

### Cell culture

Human femoral heads were generously obtained from Dr. Paul Manner (University of Washington, Seattle) from total hip arthroplasty patients with informed consent and Institutional Review Board approval. Bone marrow MSCs were isolated as described previously [16], plated in expansion medium (EM) (DMEM, 9 % lot-selected fetal bovine serum (Invitrogen, Grand Island, NY; MSC-qualified), 1 % penicillin/streptomycin/Fungizone) in 150-cm^2^ culture flasks (Corning or Nunc), and incubated at 37 °C, 5 % CO_2_. One to two days later, hematopoietic cells were rinsed away with phosphate-buffered saline (PBS) or Hank’s Balanced Salt Solution (HBSS). MSCs were routinely passaged every three to four days before reaching confluency.

To obtain OBCs, second or third passage MSCs were trypsinized and re-plated in osteogenic medium (OM) consisting of EM plus 10 mM β-glycerophosphate, 19.5 mM L-ascorbic acid-2-phosphate, 10 nM dexamethasone (all from Sigma-Aldrich, St. Louis, MO, USA), 10 nM 1,25-dihydroxyvitamin D_3_ (Enzo, Farmingdale, NY, USA) [16]. MSCs were cultured in OM for a minimum of 14 days before being considered OBCs.

### Direct co-cultures of MSCs and OBCs

To assess endogenous levels of osteoblastic mRNAs in osteogenically differentiating MSCs in contact with OBCs, MSCs and OBCs were co-cultured in a 1:4 ratio at 9,000-10,000 cells/cm^2^. Before co-culture, trypsinized MSCs were stained with the cell tracker CM-DiI (Thermo Fisher, Waltham, MA; 10^6^ cells/mL + 8 μL CM-DiI, 37 °C, for five min, then 4 °C, for 15 min) to distinguish them from OBCs and to allow subsequent fluorescence-activated cell sorting (FACS). Three co-culture combinations were tested in both EM and OM: (1) DiI-labeled MSCs mixed with unlabeled OBCs, (2) DiI-labeled MSCs mixed with unlabeled MSCs, and (3) unlabeled, MSC-only cell populations, with the latter two conditions serving as controls. Samples were harvested pre-culture, and at culture days 6 and 12. MSC-only cultures were trypsinized, centrifuged, resuspended in 1 mL Trizol (Invitrogen), and stored at -80 °C; mixed cultures were trypsinized and frozen in dimethyl sulfoxide (DMSO) freeze medium (BioVeris Corporation, Gaithersburg, MD, USA) until the time course was complete. The DiI-positive cells were fractionated using a Becton-Dickinson three-laser Dako MoFlo FACS sorter, pelleted by centrifugation, resuspended in 1 mL Trizol, and stored at -80 °C. Osteogenesis was assessed by conventional real time RT-PCR and by PCR array analysis (SuperArray, Qiagen, Valencia, CA, USA; see below).

### Conditioned medium cultures

To assess the effects of OB-secreted factors on MSC osteogenesis, EM and OM were conditioned for three days by OBCs that had completed 11-14 days of osteogenesis. Conditioned medium (CM) was frozen at -80 °C until use. OM incubated without cells for three days, referred to as aged osteogenic medium (AOM), served as the control. Thawed CM was mixed 1:1 with fresh OM before use (Note: Fresh OM was added to minimize possible medium nutrient deprivation as a confounding factor). MSCs, seeded at 8-10 × 10^3^ /cm^2^ in 6- and 12-well plates and exposed to CM every three days, were analyzed for osteogenesis, as described below.

### Matrix cultures

The effects of devitalized/decellularized OBC ECM on MSC osteogenesis were analyzed as follows: MSCs seeded at a density of 8-10 × 10^3^ cells/cm^2^ in 6-well plates were cultured in OM for at least 14 days, rinsed with HBSS, and decellularized by lysis, either with three washes of 3 mL/well MilliQ-purified water (ddH_2_O) or by one wash of 1 mL/well 0.5 % deoxycholate (Sigma-Aldrich) in ddH_2_O (DOC) for 30 min at 4 °C. The plates were then washed with HBSS three times for at least three min each time. Live/Dead staining (Invitrogen) was performed in one well of each treatment type to ensure 100 % cell death. MSCs were then seeded at 8-10 × 10^3^ cells/cm^2^ on top of the two types of matrix, with tissue culture plastic as the control, and osteogenesis was assessed as described below.

MSC proliferation was assessed fluorimetrically (494 nm/567 nm), based on calcein incorporation and Ca^2+^-dependent fluorescence, after Live/Dead staining (Invitrogen) performed on a Wallac Victor^2^ V plate reader (Perkin Elmer, Waltham, MA, USA). Nine measurements were made in a 3 × 3 pattern within each well of a 24-well plate, corrected with readings of matrix without cells.

### Real-Time RT-PCR

RNA extracted from frozen Trizol samples was reverse transcribed using oligo-d(T) and a First Strand Synthesis kit (SSII, Invitrogen) and the cDNA was amplified using SybrGreen (BioRad, Hercules, CA, USA) on an iCycler (BioRad). Primer sets (5′–3′) were designed (*see* Additional file 1: Table S1) for the following human genes: osteocalcin (OC), ALP, Runx2, collagen type I, α2 (Col I), and glyceraldehyde 3-phosphate dehydrogenase (GAPDH). Standard curves generated using a 10-fold dilution series created from a single batch of cDNA from day 18 OBCs derived from a single donor allowed relative comparisons of mRNA copy numbers between donors and experiments. Expression levels were normalized to GAPDH in triplicate.

### Microarray analysis

Human osteogenesis RT^2^ Profiler™ PCR Superarrays (SABiosciences; http://www.sabiosciences.com/PCRArrayPlate.php) were used to monitor 84 different genes associated with osteogenesis. For the matrix study, 500 ng of cDNA were loaded per plate (5 ng/well). Since FACS sorting yielded limited cell numbers in the co-culture experiments, these samples were pre-amplified by 12 rounds of PCR prior to loading them on the Superarray plates. A total of 17.5 ng of mRNA was pre-amplified using RT^2^ Nano PreAMP cDNA synthesis kits plus the corresponding NanoAmp osteogenic primers (SABiosciences) according to the manufacturer’s protocol.

The fluorescence threshold for each plate was adjusted to give an average positive PCR control (PPC) value of 20 ± 2 cycle thresholds (CTs). Transcripts whose CTs were >35 were assumed to be absent [22]. For pre-amplified samples, the cut-off CT was 30, as recommended by the manufacturer (personal communication, SABiosciences representative).

Data analysis was carried out via the SABiosciences web portal. The geometric mean of the housekeeping gene CT values was subtracted from the CT value of each gene of interest for normalization. Housekeeping genes whose CT values varied by more than two-fold among the conditions were excluded.

### Osteogenic differentiation assays

To visualize matrix mineralization, cultures were stained with 2 % Alizarin Red S (AlzR), pH 4.2 (Rowley Biochemical Institute, Danvers, MA, USA). ALP activity was monitored histochemically using the Fast Blue, leukocyte alkaline phosphatase kit (Sigma, 86R-1KT).

### Imaging

Histochemical staining was imaged at low (5X) and high (63X) magnifications, and as whole-plate scans at a resolution of 2,400 ppi. Fluorescent Live/Dead staining was examined at 10× magnification and images analyzed using AxioVision software.

### 2D-Differential in-gel electrophoresis (2D-DIGE) and protein identification

#### Sample preparation

OBCs were cultured from three different MSC donors and lysed as described for the matrix cultures. After the final rinse, matrix proteins were extracted using 7 M urea, 2 M thiourea, 30 mM Tris-HCl, 4 % CHAPS, pH 8.8 and stored at -80 °C until use. Proteins were precipitated using the 2D Clean Up Kit (GE Healthcare Bio-Sciences, Pittsburgh, PA, USA) and quantified by the 2D Quant Kit (GE), with pH adjusted to 8.0 - 9.0. A standard was created by mixing together equivalent aliquots of each sample.

#### CyDye labeling

Solutions of CyDyes 2, 3, and 5 (GE) in dimethylformamide (Sigma) were added to the samples at final concentrations of 400 pmol per 50 μg protein. The standard was labeled with Cy2 while individual samples were labeled with either Cy3 or Cy5, assigned randomly, for 30 min on ice in the dark, followed by quenching with lysine (Sigma). Samples were flash frozen and stored at -80 °C until use.

#### First dimension gel rehydration and sample loading

Thawed samples were equilibrated (1:1, v/v) with 2× sample buffer (7 M urea, 2 M thiourea, 4 % CHAPS, 4 % IPG 3-10, 6.2 mg diothiothreitol/mL) and pooled appropriately. Each mixture was added to rehydration buffer (7 M urea, 2 M thiourea, 4 % CHAPS, 2 % IPG 3-10, plus 12 μL DeStreak Solution [GE]/mL before use) for a total volume of 340 μL, and used to rehydrate an Immobiline™ 18 cm IEF DryStrip, pH 3-10 (GE) overnight.

#### Isoelectric focusing

A Multiphor II electrophoresis unit (GE Healthcare Bio-Sciences) was used with the following program at 20 °C in the dark: constant current of 1 mA; constant power of 5 W; 500 V for 0.01 h; 500 V for 3 h; gradual ramping from 500 to 3,500 V over 5 h; and 3,500 V for 12.5 h. Unless used immediately for second dimension electrophoresis, IEF gels were wrapped in plastic, placed in airtight tubes, flash frozen in liquid nitrogen, and stored at -80 °C until use.

#### SDS-polyacrylamide gel electrophoresis

The second dimension gels were 1 mm thick, 4-20 % SDS-polyacrylamide gels cast in low-fluorescence glass plates (Jule Biotechnologies, Milford, CT, USA). Electrofocused gels were incubated for 15 min in the dark in reducing buffer (0.5 M Tris-HCl, pH 6.8, 10 mg dithiothreitol/mL), followed by incubation for 15 min in alkylation buffer (10 % reduction buffer without dithiothreitol, 6 M urea, 30 % glycerol, 1 % SDS, 25 mg iodoacetamide/mL), placed on the SDS gel, and sealed with 1 % low-melt agarose. Fluorescent M_r_ standards (Sigma) were used. Electrophoresis was run at 25 °C (2 W/gel, 45 min; 17 W/gel, 3-4 h). Gels were stored moist in the dark at 4 °C overnight.

CyDye-protein fluorescence was imaged on a Molecular Dynamics Typhoon 9410 Variable Mode Imager at a resolution of 100 pixels/μm. The gel that would be used for picking protein spots (“pick gel”) was then stained with SimplySafe Coomassie blue (Invitrogen), and fixed using two to three cycles of a 15 min wash in 10 % ethanol, followed by a 15 min wash in water. The Coomassie staining of the pick gel was then imaged.

#### Protein spot comparison: DeCyder™ analysis

Gel images were analyzed using DeCyder™ (GE) to detect protein spots in each gel. Spots were excluded if they had slopes >1.3, areas <260 pixels, volumes <45,000 pixels, and peak heights <600 or >65,000 relative fluorescence units. Spots resulting from the M_r_ standards were also excluded to prevent them from being used in the statistical analysis of biological variance. The rest of the spots were analyzed manually to ensure accurate discrimination. The software then used the Cy2 standard, included in each gel, to match each protein spot to its related spots in the other gels. This matching was again checked manually and corrected, as needed. Finally, a “pick list” was generated by comparing all occurrences of each spot between gels and choosing those spots whose average volumes were > ±1.5-fold different from the standard (p ≤ 0.05).

#### Mass spectrometric identification of protein spots

Protein spots from the “pick list” were cut out of the gel in 1 mm^2^ pieces, destained, and digested overnight at 37 °C in trypsin (0.02 μg/μL in 25 mM Tris-HCl, pH 8.0) (Promega, Madison, WI, USA) to just cover the gel pieces, with extra buffer on top. Peptides were extracted with 60 % acetonitrile/3 % trifluoroacetic acid and dried.

Mass spectrometry analysis of gel digest samples was generously performed by Dr. Lewis Pannell (University of Alabama). Samples, dissolved in 15 μL 1 % acetic acid/2 % acetonitrile, were run on an ESI-TRAP mass spectrometer (ThermoElectron LTQ Orbitrap) coupled to an Agilent 1200 nano LC system with a C18 reverse phase column, eluted with 5-90 % acetonitrile (in 0.2 % formic acid) over 40 min. The acquired data files were converted into Mascot generic format (.mgf) and matched against the Swiss Protein (Sprot) *Homo sapiens* database through the MASCOT search engine (http://www.matrixscience.com). The following additional parameters were used: fixed iodoacetamidation of cysteines, variable deamidation, standard scoring, and requiring at least one unique peptide per protein match (“require bold red”). Mass accuracy for precursor ions was set to ±10 ppm and ±0.6 Da for MS/MS fragmentation data. Only proteins with at least three significant peptide matches (p < 0.05) and ion scores ≥200 were considered. Proteins that were identified in gel blanks or that matched the proteins of the M_r_ markers were excluded from further analysis.

#### Ingenuity analysis

*In silico* analysis and compilation of gene expression profiles and 2D-DIGE protein data was performed using Ingenuity Systems software (Qiagen). Changes ≥3-fold of normalized gene expression from MSCs in OM on matrix versus plastic were analyzed with their corresponding p-values (≤0.1). For each gene, the fold change at day 6 or day 12 that had the higher value and/or the greater significance was chosen to represent that gene. Proteins identified by mass spectrometry in the 2D gels were analyzed together with their maximum scores from Mascot.

### Statistical analysis

All statistical comparisons used an unpaired Student’s t-test with a p-value of ≤0.05, unless otherwise specified. Graphic portrayals of the data show either standard deviations (SD) or standard errors of the mean (SEM), as specified. Data analysis was performed using Prism (GraphPad Software, La Jolla, CA, USA), Excel (Microsoft, Redmond, WA, USA), FlowJo (Tree Star, Ashland, OR, USA), and ImageQuant (GE).

## Results

### MSC/OBC direct contact cultures

To assess the osteogenic progression of MSCs cultured in direct contact with OBCs, RT-PCR and human osteogenesis Superarray analyses were performed on FACS-sorted MSCs that had been labeled with DiI and then co-cultured with OBCs while undergoing osteogenic differentiation. We first verified that the DiI labeling did not alter MSC gene expression or morphology (Additional file 2: Figure S1). Our results showed that in mixed co-cultures, no obvious morphological differences (Additional file 3: Figure S2) nor consistent changes in gene expression (Additional file 4: Figure S3) were seen (n = 5).

### MSC Osteogenesis in OBC-Conditioned medium

To further characterize the nature of OBC-to-MSC signaling, MSCs were cultured in OM that had previously been conditioned by OBCs during days 11-14 of their osteogenic progression. MSC expression of OC, ALP, Col I and Runx2, as assayed by real time RT-PCR, was not significantly different in OBC-conditioned OM versus the AOM control (Additional file 5: Figure S4). None of the changes in gene expression were greater than 1.7-fold and none of them were statistically significant (n = 3). This was confirmed by AlzR histological staining of matrix mineralization and ALP histochemical staining (data not shown).

### Mineralization of MSCs on OBC Matrix

**Fig. 1:**
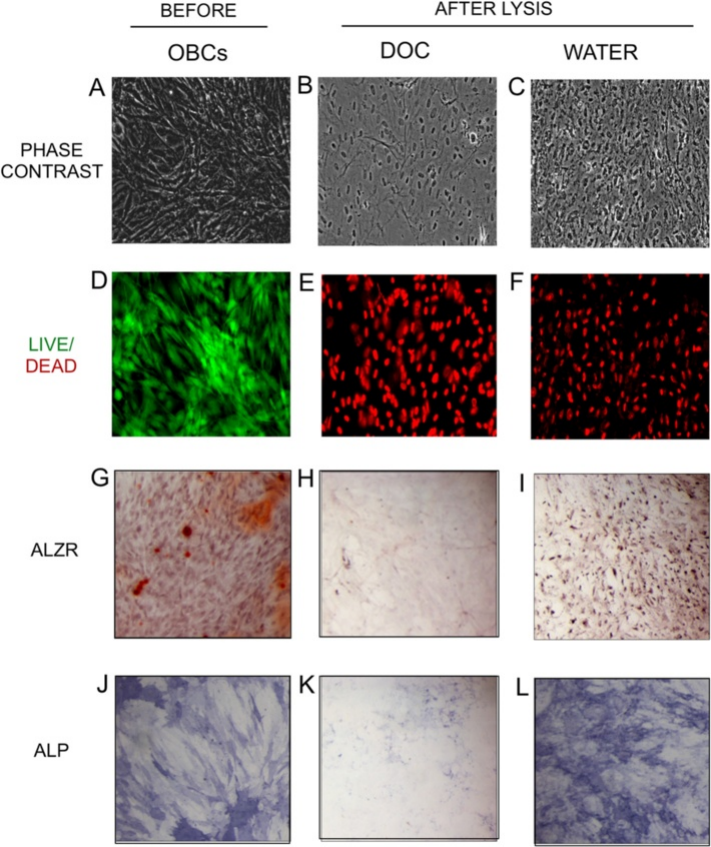
Lysis of osteoblastic cells. MSC cultures were osteogenically differentiated for 15 days, lysed with DOC or water, and examined microscopically. **a**-**c** Phase contrast images of residual matrix after lysis. **d**-**f** Fluorescent live (green)/dead (red) merged images. **g**-**i** AlzR staining of matrix calcification. **j**-**l** blue staining of ALP enzymatic activity. (**a**, **d**, **g**, **j**) OBCs before lysis. (**b**, **e**, **h**, **k**) OBCs after DOC lysis. (**c**, **f**, **i**, **l**) OBCs after water lysis. Microscopy images taken at 10×. *MSC* mesenchymal stem cells, *AlzR* Alizarin Red S, *ALP* alkaline phosphatase, *OBCs* osteoblastic cells

Nascent osteoblastic ECM was prepared by lysing OBCs with water or with 0.5 % DOC, producing matrices of distinct complexities. Water lysis was chosen because it does not denature proteins and therefore leaves the ECM mostly intact; DOC was used to decrease the complexity of the ECM by extracting some membrane proteins while being sufficiently mild to leave behind most of the ECM. While these treatments left behind predictably different amounts of matrix proteins and cellular debris (Fig. 1a-c), both lysis methods efficiently killed all OBCs (Fig. 1d-f). AlzR and ALP staining was generally depleted by both lysis methods (Fig. 1g-l), although more ALP activity was retained after water lysis (Fig. 1l).

**Fig. 2:**
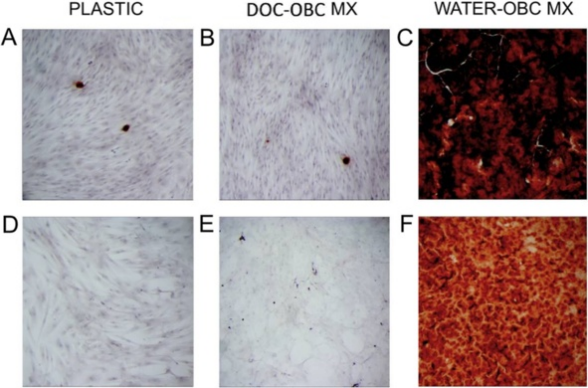
Water-treated OBC matrix enhances matrix mineralization of MSCs. OBC matrix was prepared from the cells shown in Fig. 1 at day 15 of osteogenic induction. Cultures were stained with AlzR to detect mineralization. MSCs are shown on plastic at day 12 of culture in EM (**d**) and OM (**a**). **e** DOC- and (**f**) water-treated matrices, without MSCs on top and maintained in OM for 12 days, show that water-treated OBC matrix sometimes remineralizes on its own (**f**). **b** and **c** show MSCs seeded on DOC- and water-treated OBC matrix, respectively, after 12 days in OM. DOC-matrix did not affect mineralization compared to cells on plastic (**a**). While water-treated matrix alone (**f**) showed a high level of AlzR staining even in the absence of live cells, the presence of MSCs enhanced staining (**c**). *OBC* osteoblastic cells, *MSC* mesenchymal stem cells, *AlzR* Alizarin Red S, *EM* expansion medium, *OM* osteogenic medium

**Fig. 3:**
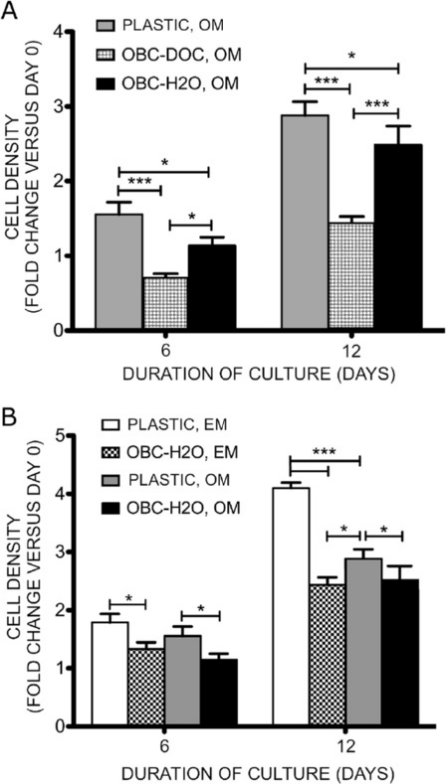
OBC matrix slows MSC proliferation in EM and OM. MSCs were plated on plastic or DOC-treated or water-treated OBC matrix and maintained in OM or EM. Their proliferation was examined on culture days 6 and 12. **a** In OM, MSC proliferation was reduced by 2-fold on DOC-treated OBC matrix and 1.2-fold on water-treated matrix. **b** MSC proliferation on plastic was 1.3-fold lower in OM than in EM, but this reduced proliferation is absent when comparing cells in EM and OM on water-treated OBC matrix. Fold changes are compared to the pre-culture value and expressed as mean ± SEM. Data are from a single experiment, representative of three experiments. *, p < 0.5, **, p < 0.01, ***, p < 0.001. *OBC* osteoblastic cells, *MSC* mesenchymal stem cells, *EM* expansion medium, *OM* osteogenic medium, *SEM* standard error of the mean

To assess the effects of OBC matrix on MSC osteogenesis, MSCs were seeded on top of both types of matrix. MSCs on water-treated (but not DOC-treated) matrix increased their mineralization compared to those seeded on plastic, as shown by AlzR staining (Fig. 2a-c). While water-treated OBC matrix could mineralize heavily in OM without live MSCs on top (Fig. 2f), possibly due to the presence of matrix calcium binding proteins, such as osteocalcin, the mineralization seen with MSCs present was substantially more intense (Fig. 2c). This increase was not due to an increase in MSC proliferation on matrix. In fact, calcein fluorescence staining showed that MSCs proliferated slightly less on water-treated OBC matrix than on plastic, and they proliferated very little when plated on DOC-treated matrix (Fig. 3a). As expected, MSC proliferation was lower in OM than in EM, but, interestingly, this decrease was not apparent when MSCs were plated on water-treated matrix (Fig. 3b).

### Gene expression and morphology of MSCs on decellularized OBC matrix

**Fig. 4:**
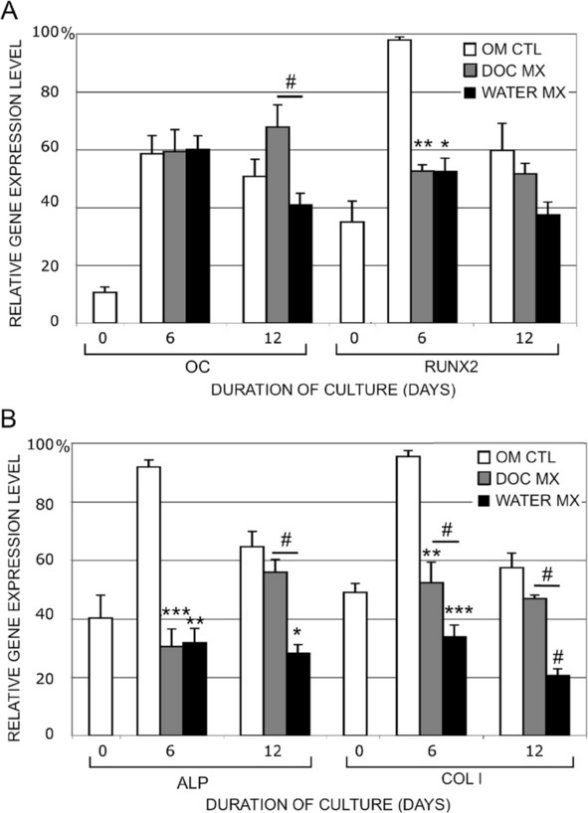
OBC matrix suppresses MSC expression of osteogenic genes. MSCs were seeded on top of OBC matrix (treated with water or DOC) and cultured in OM for 6 and 12 days. Gene expression of **a **OC, Runx2, **b **ALP, and ColI was assayed by real-time RT-PCR. Expression levels were normalized to GAPDH and shown as a percentage of the maximum level within each experiment. Values are means ± SEM of five experiments. Asterisks indicate significance between experimental conditions and the OM control. Bars show significance between matrix conditions. #, p < 0.1, *, p < 0.05, **, p < 0.01, ***, p < 0.001. *OBC* osteoblastic cells, *MSC* mesenchymal stem cells, *OM* osteogenic medium, *RT-PCR* reverse transcription-polymerase chain reaction, *SEM* standard error of the mean

Although our initial observations based on increased AlzR staining suggested that OBC matrix could enhance MSC osteogenesis, gene expression analysis suggested otherwise. Real time RT-PCR analysis of three key osteoblastic genes, Runx2, ALP, and Col I, consistently showed down-regulation in MSCs seeded on OBC matrix versus plastic (n = 5) (Fig. 4). Only OC expression showed no significant differences between plastic and DOC- or water-treated matrix cultures (Fig. 4a). Runx2 levels dropped by nearly 50 % on both matrices at day 6 of culture. While these large decreases were not maintained through day 12, the downward trend remained. ALP levels were only one-third as high on both matrices as on plastic at day 6. Day 12 ALP levels on DOC matrix were similar to those on plastic but levels on water matrix remained at less than 50 % of those on plastic. Finally, Col I levels at day 6 dropped by 50 % on DOC matrix and by 70 % on water matrix. At day 12, Col I levels on DOC matrix were similar to those on plastic, while those on water matrix were again only one-third as high as those on plastic (p = 0.1). It is noteworthy that for several genes, there was a trend suggesting that water matrix suppressed osteogenic gene expression to a greater extent than DOC matrix: Col I at day 6; OC, ALP and Col I at day 12 (p < 0.1) (Fig. 4).

To confirm these data and broaden the number of genes being assessed, we carried out real-time RT-PCR microarray analysis using human osteogenesis Superarrays (*see* Additional file 6: Figure S5.pdf). Changes ≥3-fold were analyzed. The trends seen previously in the four osteogenic markers (above) were somewhat altered, presumably due to donor variability. Changes in ALP and Runx2 levels did not reach three-fold, although ALP levels were reduced by more than two-fold on water-matrix on days 6 and 12 and on DOC-matrix on day 6. Runx2 levels decreased by 2.3-fold on DOC-matrix on day 6 but all other conditions elicited little change. Twenty-one genes related to osteogenesis displayed ≥3-fold changes in MSCs on matrix, including OC and Col I. All of them were down-regulated except CSF2, CSF3, ITGA2, OC and VEGFA. OC was up only on day 6 in MSCs on DOC-matrix. The down-regulated genes included BGN, BMP4, CD36, CDH11, COL1A1, COL1A2, COL4A3, COL10A1, COL11A1, COL12A1, COMP, FGFR2, ICAM1, IGF1, IGF2, and MMP8. Overall, while the Superarray data did not reflect the greater potency of water-treated matrix versus DOC-treated matrix, suppression of MSC osteogenesis by OBC matrix was confirmed.

**Fig. 5:**
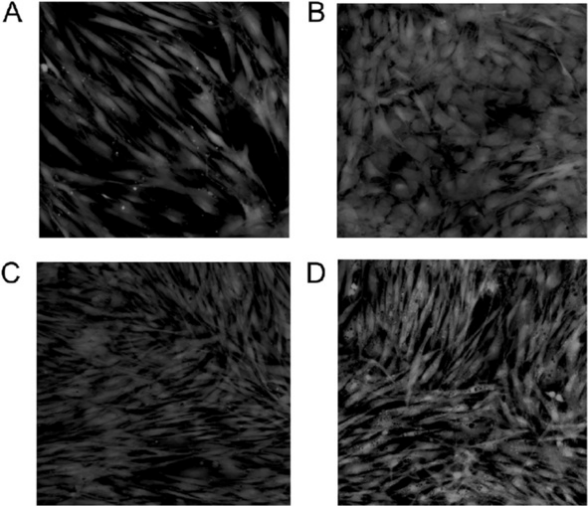
OBC matrix preserves the naïve, fibroblastic morphology of MSCs. Fluorescence microscopy of Calcein AM-labeled cells shows that, even at day 12, MSCs cultured in OM and seeded on OBC matrices (**c**, DOC- and **d**, water-treated) retain their fibroblastic morphology, like MSCs cultured in EM on plastic (**a**), instead of becoming cuboidal, like cells cultured in OM on plastic (**b**). **a, b** Viewed at day 6. *OBC* osteoblastic cells, *MSC* mesenchymal stem cells, *OM* osteogenic medium, *EM* expansion medium

In support of this observed reduction in osteogenic gene expression, we also noted that the MSCs cultured on OBC matrix did not assume an osteoblastic morphology. On plastic, MSCs showed a marked transformation in shape from long and fibroblastic in EM (Fig. 5a) to bunched and cuboidal in OM (Fig. 5b). However, when these same cells were cultured in OM on either DOC- or water-treated OBC matrix, their morphology remained fibroblastic even through day 12 (Fig. 5c and d, respectively).

### Specificity of the effect of OBC-derived matrix

Finally, it should be noted that the observed effects of OBC matrix on MSC osteogenesis appeared to be OBC-specific. This was confirmed by comparing the effect of OBC matrix on MSC osteogenesis to that of matrix derived from human foreskin fibroblasts (HFF), prepared using an identical water lysis decellularization protocol. As shown in Additional file 7: Figure S6, HFF-derived matrix did not suppress the ALP level as effectively as OBC-derived matrix.

### OBC matrix protein identification by 2D-DIGE

**Fig. 6:**
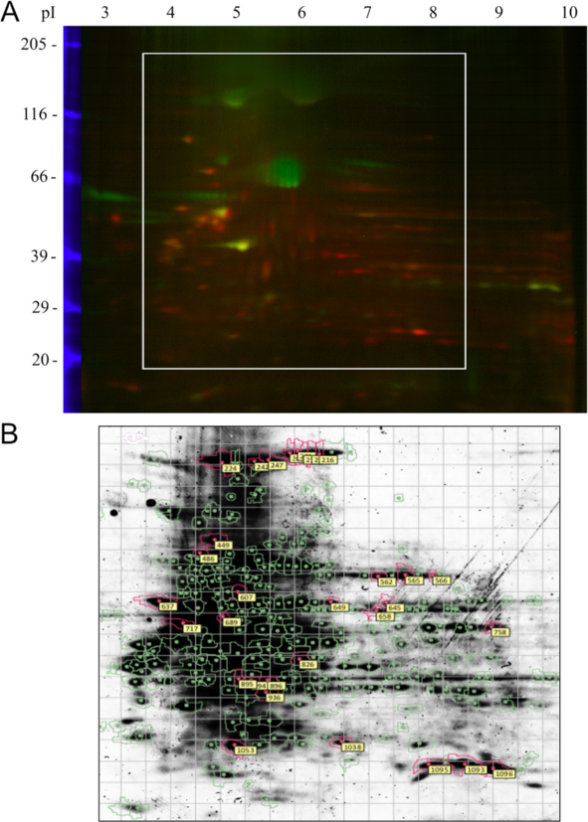
2D-DIGE analysis of OBC matrix proteins. **a** A fluorescent image of a representative 2D gel. Blue bands at *left* represent fluorescent M_r_ standards. DOC-treated OBC matrix proteins were labeled with Cy3 (green); water-lysed OBC matrix proteins with Cy5 (red). The linear, immobilized IEF gradient gel is at the top with the acidic end (pH 3) at the *left* and the basic end (pH 10) at the *right*. The *white box* shows the approximate area of the Coomassie-stained gel shown in (**b**) from which the protein spots were excised. Excised spots are circled in pink and labeled with a spot number. *2D-DIGE* 2D-differential in-gel electrophoresis, *OBC* osteoblastic cells, *IEF* isoelectric focusing gel

**Fig. 7:**
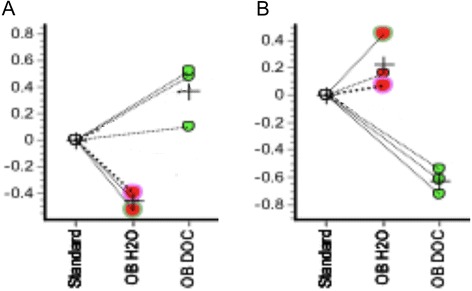
Graphs showing the relative abundance of matrix proteins from different spots. **a** Spot 449. **b** Spot 1096

**Fig. 8:**
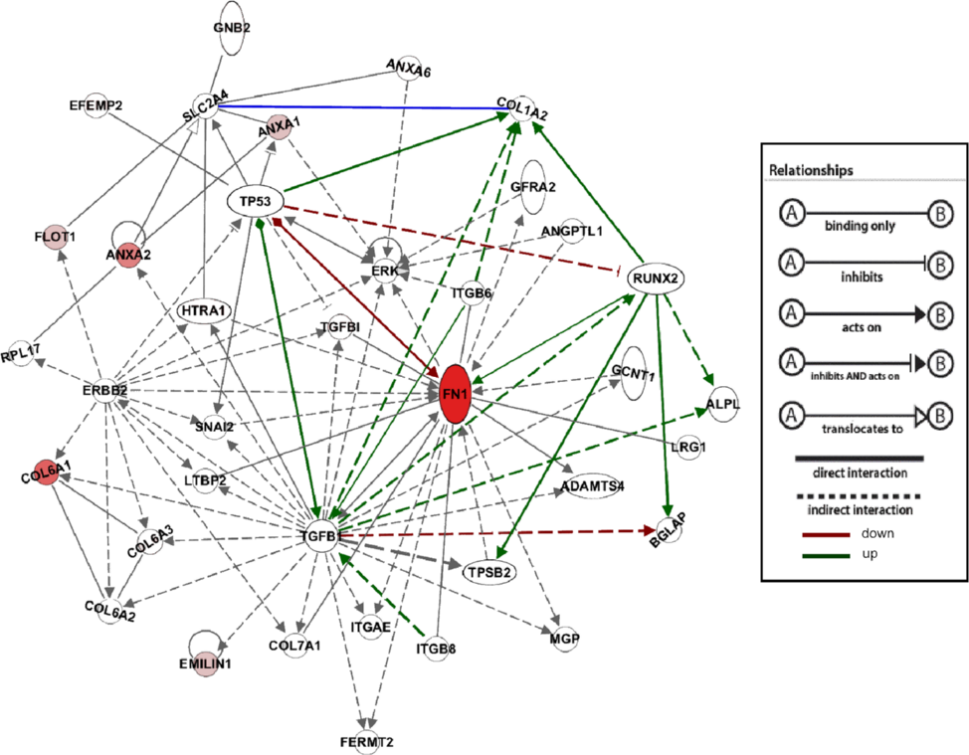
Connections to regulators of osteogenesis and cellular proliferation exhibited by proteins identified by 2D-DIGE. This network, generated by Ingenuity®, portrays relationships described in the literature between all of the proteins identified by 2D-DIGE. Runx2 was added to emphasize the participation of these proteins in the osteogenic pathway. The proteins from the gel spots are colored in varying shades of red relative to the match score they received from Mascot. Together with TGF-β1, Runx2 and TP53 are hubs for regulation of osteogenesis and cellular proliferation. (Note: *Gray arrows* indicate action, not up/down-regulation. Only lines leading to and away from the four focus genes are color-coded.) Protein symbols are listed in (Additional file 8: Table S2). *2D-DIGE* 2D-differential in-gel electrophoresis

To begin identifying the proteins responsible for the observed effects on MSC osteogenic differentiation, proteins found in DOC and water matrix were compared through 2D-DIGE (Fig. 6). The amount of each protein from three samples of each matrix type was compared to the protein standard contained within each gel. Their relative amounts were plotted on graphs (Fig. 7) and compared. Table 1 shows the “pick list”, the 29 gel spots found by DeCyder to be at least 1.5-fold more abundant in one matrix versus the other (p < 0.05). Table 2 lists the proteins identified by mass spectrometry from the gel spots on the “pick list.” The most likely candidate proteins, due to their known extracellular location, are those of the ECM, including collagen type VI α1 and α3 (COL6A1, COL6A3), elastin microfibril interfacer (EMILIN1), EGF-containing fibulin-like extracellular matrix protein (EFEMP2), heat shock-induced serine protease HTRA1, and TGF-β-induced protein (TGFBI). Several plasma membrane proteins are also strong candidates, including annexins A1, A2 and A6 (ANXA1, ANXA2, ANXA6), flotillin (FLOT1), and fibronectin I (FN1). COL6A1 and A3, EMILIN1, and FN1 were identified in spots that were more intense in DOC-treated matrix, while the others plus COL6A3 were more abundant in water-treated matrix. (COL6A3 was present in more than one protein spot.) These 11 proteins were organized by Ingenuity into functional groups. Groups for cell morphology, cellular assembly and organization, cellular growth and proliferation, cell-to-cell signaling and interaction, and cellular movement each included at least five of the focus proteins and received p-values of <0.05. Their putative relationships with each other, and with the osteoblastic genes RUNX2, OC (BGLAP), COL1A2, and ALP(L), are shown in Fig. 8 (also see Additional file 8: Table S2) . As few additional proteins as possible were added (by Ingenuity) to allow each protein of interest to have at least one connection to the network.

**Table 1:** “Pick List” of statistically significant protein spots for identification

Spot No.	Avg. ratio	p-value (t-test)	Spot Vol.	pI	M_r_	Match quality
201	5.32	0.019	N/A	5.65	133,581	1.80
203	5.69	0.015	1,809,098	5.72	133,294	2.38
208	6.21	0.015	1,387,720	5.81	132,723	1.78
210	7.48	0.015	2,038,969	5.90	131,588	0.74
214	7.7	0.015	2,700,438	6.00	130,462	0.52
216	7.72	0.015	2,774,451	6.09	129,903	0.47
224	5.93	0.015	11,334,140	4.82	127,965	2.77
242	6.59	0.019	1,651,536	5.24	121,536	3.41
247	4.71	0.015	2,109,428	5.39	120,497	5.05
449	7.28	0.019	1,501,211	4.76	82,040	1.30
486	−1.6	0.038	1,347,174	4.59	75,936	2.60
562	−3.64	0.026	1,435,516	6.56	66,327	2.55
565	−3.31	0.030	2,230,394	6.85	66,184	1.05
566	−1.73	0.043	258,223	7.15	66,184	2.82
607	−2.65	0.015	1,380,551	5.02	61,260	3.09
637	−2.67	0.036	4,584,544	4.16	57,933	0.77
649	−4.96	0.015	746,894	6.09	57,561	12.13
658	−1.52	0.031	773,745	6.43	56,824	3.03
689	−1.73	0.049	3,281,833	4.82	54,319	0.67
717	−2.11	0.041	5,864,175	4.42	51,923	0.70
758	−2.09	0.041	750,800	7.99	48,788	3.34
826	−2.69	0.034	2,653,198	5.71	42,890	2.37
894	−1.93	0.015	1,912,332	5.17	38,606	5.09
895	−1.83	0.041	694,589	4.97	38,275	2.33
896	−3.09	0.015	5,361,677	5.35	38,440	2.96
1053	−2.63	0.036	2,984,453	4.99	27,203	1.65
1093	−7.66	0.015	6,128,338	7.30	24,017	2.46
1095	−4.59	0.023	1,765,854	6.92	23,914	1.91
1096	−7.55	0.015	13,937,178	7.60	23,812	0.52

DeCyder-generated list of proteins whose abundances vary by at least ±1.5-fold (p ≤ 0.05) between DOC- and water-treated OBC matrices. Columns show (left to right): numbers assigned to each spot on the gel; average ratios between spot volumes (DOC/water); p-values corresponding to those ratios; maximum spot volumes (pixels); approximate pIs and M_r_ (Daltons) based on position within the gel; and match quality, a measure of consistency between a spot’s replicates in different gels (lower is better). Spots above the double line are more abundant in DOC matrix, below, in water matrix

*OBC* osteoblastic cell

**Table 2:** Identities of Proteins from "Pick List"

	Symbol	Entrez Gene Name		Found in Spots:	Mx	Max Score	pI	M_r_
CYTOPLASM	ACTA2	**actin, alpha 2, smooth muscle, aorta**		826, 894–6, 1053	W	348	**5.23**	**42,381**
	ALDH1B1	**aldehyde dehydrogenase 1 family, member B1**		658	W	283	**6.36**	**57,658**
	AKR1C1	aldo-keto reductase family 1, member C1		894	W	240	8.02	**37,221**
	ATP5A1	ATP synthase, H+ transport, mitoch F1 complex, alpha 1		658	W	867	9.16	**59,828**
	ATP5B	**ATP synthase, H+ transport, mitoch F1 complex, beta**		689	W	683	**5.26**	**56,525**
	CKAP4	cytoskeleton-associated protein 4		717	W	821	5.63	66,097
	ENO1	**enolase 1, alpha**		758, 759	W	532	**7.01**	**47,481**
	ENO3	**enolase 3, beta, muscle**		758	W	246	**7.59**	**47,299**
	EIF3B	**eukaryotic translation initiation factor 3, subunit B**		896	W	213	**5.38**	**36,878**
	FH	fumarate hydratase		758, 759	W	290	8.85	**54,773**
	HSP90B1	**heat schock protein 90kDa beta (Grp94), member 1**		449, 636, 637	B	306	**4.76**	**92,696**
	HSPB1	**heat shock 27kDa protein 1**		1053	W	315	**5.98**	**22,826**
	HSPD1	heat shock 60kDa protein 1 (chaperonin)		607	W	390	5.83	**60,813**
	HSPA5	**heat shock 70kDa protein 5 (glucose-regulated)**		449, 486	B	1483	**5.07**	**72,402**
	IKIP	IKK interacting protein		717	W	487	9.21	39,399
	PRDX1	peroxiredoxin 1		1093	W	363	8.27	**22,324**
	P4HB	**prolyl 4-hydroxylase, beta polypeptide**		201, 607, 636	B	1116	**4.76**	**57,480**
	PSMD13	**proteasome (prosome, macropain) 26S subunit, 13**		826	W	219	**5.71**	**42,872**
	PDIA3	**protein disulfide isomerase family A, member 3**		647-9	W	706	**5.98**	**57,146**
	PDIA6	protein disulfide isomerase family A, member 6		689	W	395	5.35	**48,207**
	PDHB	pyruvate dehydrogenase (lipoamide) beta		896	W	202	6.20	**39,550**
	PKM2	pyruvate kinase, muscle		607	W	341	7.96	**58,470**
	SOD2	superoxide dismutase 2, mitochondrial		1093-6	W	347	8.35	**24,878**
	TUBA1A	**tubulin, alpha 1A**		689, 894	W	494	**4.94**	**50,788**
ECM	COL6A1	**collagen, type VI, alpha 1**		201, 203, 208, 210, 224, 242, 247	D	1432	**5.26**	**109,602**
	COL6A3	collagen, type VI, alpha 3		247, 607, 649	B	321	6.26	345,163
	EFEMP2	**EGF-containing fibulin-like ECM protein 2**		636	W	297	**4.79**	**51,725**
	EMILIN1	**elastin microfibril interfacer 1**		224-6, 242, 247	D	462	**5.07**	**107,913**
	HTRA1	HtrA serine peptidase 1		637	W	267	8.09	**52,167**
	TGFBI	transforming growth factor, beta-induced, 68kDa		486	W	247	7.62	**75,261**
PM	ANXA1	annexin A1		894-6	W	397	6.57	**38,918**
	ANXA2	annexin A2		894-6	W	1201	8.44	**36,950**
	ANXA6	annexin A6		486	W	265	5.42	**76,168**
	FN1	fibronectin 1		all but 1093-96	D	2242	**5.45**	266,034
	FLOT1	**flotillin 1**		758	W	496	**7.08**	**47,554**
NU	CRYAB	**crystallin, alpha B**		1093-6	W	244	**6.76**	**20,146**
	LMNA	**lamin A/C**		561-66	W	1179	**6.57**	**74,380**

Columns show (left to right): the number(s) of the spot(s) where a given protein was found; the type of matrix in which the protein was more abundant (D = DOC, W = water, B = both); maximum Mascot identity score from mass spectrometry; and theoretical pIs and M_r_ values determined by Mascot from the protein sequences. Bolded text indicates that the approximate pI and/or M_r_ of the spot in which the protein was found match its theoretical values. Proteins are grouped by subcellular location based on Ingenuity’s database

*NU* nucleus, *ECM* extracellular matrix, *PM* plasma membrane

## Discussion

In this study, we have attempted to model the bone marrow niche of adult MSCs in vitro by generating a nascent osteoblast environment and testing its effect on MSC osteogenesis. When RT-PCR was used after FACS separation of cocultured MSCs, no differences were detected in osteogenic transcript levels between MSCs cultured with and without contact with OBCs. These results are corroborated by the findings of Wang et al. [15] in a similar study using mouse cells. These authors mixed MC3T3-E1 mouse osteoprogenitor cells with MSCs from GFP-transgenic mice, cultured them in OM at a plating density of 5,000/cm^2^ for 21 days, then FACS sorted the MSCs based on green fluorescent protein (GFP) fluorescence, and analyzed the transcript levels of osteogenic genes. Their results showed that direct cell-cell contact with the osteoblastic cells did not significantly alter the osteogenic differentiation of the MSCs.

As in our study, Wang et al. [15] also examined the effects of OB-secreted proteins on the osteogenesis of MSCs. When mouse MSCs were cultured indirectly with MC3T3 cells in OM using Transwell inserts, the proliferation of neither cell type was affected, but matrix calcification was enhanced and several osteogenic gene transcript levels were significantly increased. The differences in our results may be related to the developmental stage of the OBs used: the mouse MC3T3 osteogenic cell line is derived from newborn mouse calvaria, while the OBCs used here are recently derived from adult human MSCs. It is also known that mouse and human MSCs do not respond equivalently to all osteogenic stimuli [23]. Additionally, other studies have also reported a lack of osteo-induction by OB-secreted factors. For example, Gerstenfeld et al. [11] found that C3H10T^1^/_2_ mouse embryonic MSCs did not increase their expression of OC when co-cultured in Transwells with chick embryonic calvarial OBs.

A number of studies have assessed the effects of various ECMs and their individual components on the osteogenic differentiation of mesenchymal cells [24, 25]. Although the experimental systems were usually simplified to examine the effects of a single ECM protein on MSC osteogenesis, many of the studies reported conflicting results. Shi et al. [21] found that Col I-coated plates stimulated Col I, ALP and osteopontin gene expression in rat neonatal calvarial cells (ROB-C26) without osteo-inductive medium, but the Col I matrix was not sufficient to induce ALP activity or the expression of TGF-α1, TGF-β2 or BMP-6. Salasznyk et al. [26] concluded similarly that human MSCs can be induced to differentiate into OBs through contact with Col I or vitronectin, without osteogenic supplements, as assessed via mineralization and Col I, OPN and ALP mRNA. Cool and Nurcombe [27], however, saw no differences in proliferation, ALP activity or Runx2, Col I or OC mRNA levels in murine MSCs seeded on Col I, FN1 or laminin substrates and cultured in OM. (Note: culturing in EM was not tested.) Nor did Heckmann et al. [28] observe an induction of OC or Col I expression when human MSCs were cultured on a three-dimensional Col I matrix. There is thus no obvious consensus in the literature regarding the effects of any single ECM protein on MSC osteogenesis.

While these apparent conflicts may be related to differences of species, substrates or culture media, these studies by their very nature are overly reductionistic in terms of mimicking the in vivo environment since the ECM is a complex mixture of multiple components, deposited in temporospatially specific configurations and proportions that are important for their biological activities. To better approximate the in vivo niche, we chose to examine the activity of the matrix laid down by nascent osteoblastic cells. The native configuration of the OBC ECM is preserved by keeping it on the original culture dishes, without decalcification, fixation, or extraction. In this context, it is instructive to review the classic work of Urist on demineralized bone matrix (DBM) [20]. In Urist’s studies, adult human and rabbit bones were lyophilized and decalcified and implanted into various muscles and bone defects. The DBM induced new bone formation within the rings of decalcified matrix. However, a later study showed that the principal inducers responsible for DBM-induced osteogenesis were BMPs sequestered in the bone matrix [29]. This was further verified by Becerra et al. [30] who found that DBM could also enhance MSC osteogenesis indirectly through Transwell inserts. The fundamental difference between DBM and the ECM used here is one of age and structure. As bone matures over time, growth factors, such as BMPs, are sequestered within the bone matrix, particularly in the mature trabecular and cortical bone, as new layers of ECM are deposited and mineralized. The new matrix used here, synthesized by nascent OBCs and grown on a two-dimensional surface, is less likely to entrap growth factors. It more closely mimics that laid down by bone-lining cells at the endosteal surface of the bone marrow cavity, i.e., it is similar to the osteoblastic matrix that MSCs likely encounter in vivo. Enhancement of MSC osteogenesis seen with mature bone matrix likely mimics what MSCs experience during fracture healing when trabecular or cortical bone is exposed along with its matrix and bound growth factors [31, 32]. MSCs, recruited to the site, thus come into contact with the growth factor-laden mature bone, which then induces them to begin osteogenic differentiation to repair the fracture.

One other study elegantly demonstrates the importance of the maturity of the OB matrix being studied [33]. ECM was produced by neonatal rat calvarial cells (ROB-C26) cultured with or without retinoic acid, which induces osteogenesis in these cells. ECM made by the more osteoblastic cells was able to induce osteogenesis in naïve C26 cells in vitro and in vivo. However, ECM synthesized by C26 cells in the absence of retinoic acid was only able to partially stimulate osteogenesis in vitro and not at all in vivo.

Furthermore, Chen et al. [34] showed that ECM from murine MSCs themselves enhanced MSC proliferation and slowed their spontaneous osteogenic differentiation, while enhancing their tri-lineage plasticity. A second study confirms this in human MSCs and identifies several of the proteins present in the ECM (Col I, Col II, FN, byglycan, decorin, perlecan, and laminin) [35].

The fact that MSCs’ own matrix can help maintain their stemness [35] is particularly interesting, not only because of its implications for the MSC niche, but also because it suggests that “concentrations” of ECM are important. Because MSC ECM is always available to MSCs, if this contact were sufficient to maintain their stemness, they would have to halt production of ECM proteins and withdraw from their ECM before differentiating. While this could be one explanation, there could also be thresholds of MSC-ECM interactions that must be reached before certain effects occur. Indeed, a similar idea was proposed by Volloch and Olsen [36] regarding the effect that the “density” of integrin-binding sites within various matrices has on MSC differentiation.

In addition to these quantitative thresholds of interacting ECM, apparently achieved in the OBC matrix studied here, there are likely qualitative characteristics of the OBC matrix, i.e., specific components that are important for the control of the differentiation activities of MSCs. Therefore, we compared the protein compositions of the DOC- and water-lysed OBC matrices using 2D-DIGE. Eleven focus proteins were chosen because of their characterization as ECM or membrane proteins, which makes them most likely to be in contact with MSCs *in vivo*. These were FN1, COL6A1, COL6A3, ANXA1, ANXA2, ANXA6, HTRA1, flotillin (FLOT1), EMILIN1, EFEMP2, and TGFBI. Several of these proteins have already been implicated in stem cell functions or osteogenesis. FN1, for example, was found in the MSC ECM reported in the study mentioned above to suppress MSC osteogenesis [34]. It has also been used to coat tissue culture dishes to enhance the proliferation and plasticity of a subpopulation of MSCs [5, 37]. ANXA1 and 2 are up-regulated in amputated tadpole tails undergoing regeneration [38]. Interestingly, they were also both identified in a proteomic analysis of mouse embryonic fibroblasts, which are the feeder layers cells used to maintain embryonic stem cells in vitro [39].

TGFBI binds collagen types I, II and IV and is expressed in most tissues [40]. It has been shown to be highly expressed in HSCs that adhere well to MSCs [41]. This observation is significant because the binding of HSCs to MSCs has been implicated in the maintenance of the HSC phenotype in culture [42]. In addition, when TGFBI is exogenously added to periodontal ligament cells, it can inhibit their mineralization, suggesting an anti-osteogenic function [43]. Recent research shows that HTRA1 may play a similar role since it inhibited mineral deposition in immortalized murine OBs (2 T3 cells) even in the presence of BMP-2 [44]. The same study also showed that its overexpression reduced levels of Runx2 and Col I mRNA. However, further research, using human MSCs, showed an opposite effect for HTRA1 [45], although it did confirm that HTRA1 secretion from MSCs increases as they undergo osteogenesis and, in mice, it is found mostly in areas of new bone formation after fracture.

EMILIN1 has not yet been linked to bone tissues but is known to bind pro-TGF-β, a key regulator of osteogenesis, in the extracellular space of blood vessels [46]. This binding inhibits the maturation of TGF-β, the purpose of which may be the prevention of fibrosis due to Col I buildup [46].

To date, several studies have shown that ECMs generated by various cell types in vitro, including MSCs themselves, can encourage MSC stemness and suppress their differentiation [34, 47–50]. It is unlikely that there is a single MSC niche produced by a single cell type. Indeed MSCs are found throughout the body in various tissues. To discover which proteins are most important in maintaining and expanding MSCs, one approach would be to compare the proteins present in OBC ECM to those found in other stemness-enhancing ECMs. Since the ECM generated by MSCs of older mice has been shown to be less efficient in maintaining their stemness [51], we suggest that the protein makeup of ECMs from human donors of various ages be compared as well.

## Conclusions

In summary, our study has shown an osteo-inhibitory function for OBC matrix laid down in vitro by nascent osteoblasts derived from osteogenically differentiated MSCs. The OBC matrix is likely to be similar to that laid down by newly-formed, active OBs that line the endosteal surface in the bone marrow milieu. Since only MSC osteogenesis is examined here, whether the “niche effect” seen is specific to osteogenesis only or applicable to the maintenance of MSC stemness in general remains to be investigated. In either case, it may be that MSCs in the marrow stroma are often in contact with similar nascent OB matrix and are thus kept in an undifferentiated state. In a traumatic bone injury setting, MSCs would be called forth to migrate from their niche to the site of fracture where they would come into contact with mature bone and its matrix, which would then act to stimulate their osteogenic differentiation via matrix-entrapped growth factors. This model, if proven correct, will strongly suggest that the OBC matrix and/or its active components may be applied in vitro to develop a means to extend the proliferative potency and therapeutic utility of MSCs by suppressing their default osteogenic differentiation.

## Additional files

Additional file 1: Table S1. Primer sequences of genes analyzed by real-time RT-PCR for osteogenesis. (DOCX 50 kb) Additional file 2: Figure S1. DiI labeling does not affect MSC osteogenic gene expression. MSCs were labeled with DiI and cultured in EM or OM for 21 days. Osteocalcin (OC) mRNA expression was measured by real-time RT-PCR and normalized to GAPDH. Unlabeled MSCs were used as controls. Values shown are mean ± SD from cells isolated from a 61-year-old male donor, plated at 3,000/cm^2^. (JPEG 305 kb) Additional file 3: Figure S2. Morphology of DiI-labeled MSCs in co-culture with unlabeled OBCs. Images of MSCs on culture days 6 and 12 show no obvious morphological differences due to co-culture with OBCs. Shown are unlabeled MSCs in EM; DiI-labeled MSCs mixed with unlabeled MSCs in OM as a control (MSC/MSC*); and DiI-labeled MSCs mixed with unlabeled OBCs in OM (OBC/MSC*). MSCs were derived from a 73-year-old male donor, plated at 9,000 cells/cm^2^ and cultured in a 1:4 ratio with OBCs induced from the same MSCs for 15 days prior to co-culture. MSC* indicates MSCs labeled with DiI (red). 10× magnification. (JPEG 1025 kb) Additional file 4: Figure S3. Effects of OBC co-culture on MSC osteogenic gene expression is variable. mRNA levels of osteogenic genes (OC, Runx2, ALP, and Col I) in MSC control cultures and MSC/OBC co-cultures were measured via real-time RT-PCR, and normalized to GAPDH. Each value was then expressed as a percentage of the maximum level reached for that gene in cells from a given donor within a given experiment. Values shown are the mean ± SEM from two experiments. No statistically significant differences were observed. (JPEG 238 kb) Additional file 5: Figure S4. Minimal effects of OBC-conditioned medium on MSC osteogenic gene expression. mRNA levels of OC, Runx2, ALP, and Col I measured via real-time RT-PCR (normalized to GAPDH) were compared between MSCs in OBC-conditioned OM (CM) versus those in the control aged OM (AOM) cultured for 3, 6 and 12 days. Mean fold changes on each day from three experiments are shown (mean ± SD). None of the changes in gene expression upon exposure to OBC-CM were greater than 1.7-fold, and none of them were statistically significant. (JPEG 147 kb) Additional file 6: Figure S5 Microarray analysis confirms the suppression of osteogenesis by OBC matrices. MSCs from three donors were cultured in OM on OBC matrices prepared by water or DOC lysis, and gene expression analyzed by real-time RT-PCR using the SuperArray System. Gene-of –interest CT values were normalized to the average of five housekeeping genes. Shown are those genes that showed mean fold changes > 3-fold when comparing MSCs on matrix to MSCs on plastic in OM at days 6 and 12. Genes were assigned to graph (A) or (B) based on alphabetical order. Asterisks indicate significance between an experimental condition and the OM plastic control. There was no statistical significance in any differences >3-fold between matrix treatments. *, p < 0.05; **, p < 0.01. (JPEG 427 kb) Additional file 7: Figure S6. Comparison of the matrix effect of human foreskin fibroblasts and osteoblastic cells on osteogenic differentiation of MSCs. HFF and OBC cultures were water-lysed and the resultant matrices used as a substrate for MSCs cultures maintained in osteogenic medium. ALP activity was detected histochemically. Non-MSC seeded HFF and OBC matrices were used as control. The results showed that HFF matrix did not suppress MSC osteogenesis, compared to OBC matrix. OBCs were derived from day-15 osteogenic culture of bone marrow derived MSCs from a 47-year-old female donor. (JPEG 637 kb) Additional file 8: Table S2. Protein symbols used for proteomic analysis. (DOCX 122 kb)
