# Supplementary material for: Nascent osteoblast matrix inhibits osteogenesis of human mesenchymal stem cells in vitro
Source: Stem Cell Res Ther. 2015 Dec 22;6:258. doi: 10.1186/s13287-015-0223-x (PMC4688995; doi:10.1186/s13287-015-0223-x)
Supplement: Additional file 1: Table S1. — Primer sequences of genes analyzed by real-time RT-PCR for osteogenesis. (DOCX 50 kb) [file 13287_2015_223_MOESM1_ESM.docx]

Table S1

Primer Sequences of Genes Analyzed by Real-time RT-PCR for Osteogenesis

| Gene | Sense Primer | Antisense Primer |
| --- | --- | --- |
| OC | AGGAGGGCAGCGAGGTAG | GAAAGCCGATGTGGTCAGC |
| ALP | TCAGAAGCTCAACACCAACG | GTCAGGGACCTGGGCATT |
| Runx2 | GCGGAAGCATTCTGGAA | ACTGGGCCCTTTTTCAGA |
| Col Iα2 | GGTGTAAGCGGTGGTGGTTAT | GCTGGGATGTTTTCAGGTTGG |
| GAPDH | CAAGGCTGAGAACGGGAAGC | AGGGGGCAGAGATGATGACC |
