# Supplementary figures and images for: Nascent osteoblast matrix inhibits osteogenesis of human mesenchymal stem cells in vitro
Source: Stem Cell Res Ther. 2015 Dec 22;6:258. doi: 10.1186/s13287-015-0223-x (PMC4688995; doi:10.1186/s13287-015-0223-x)

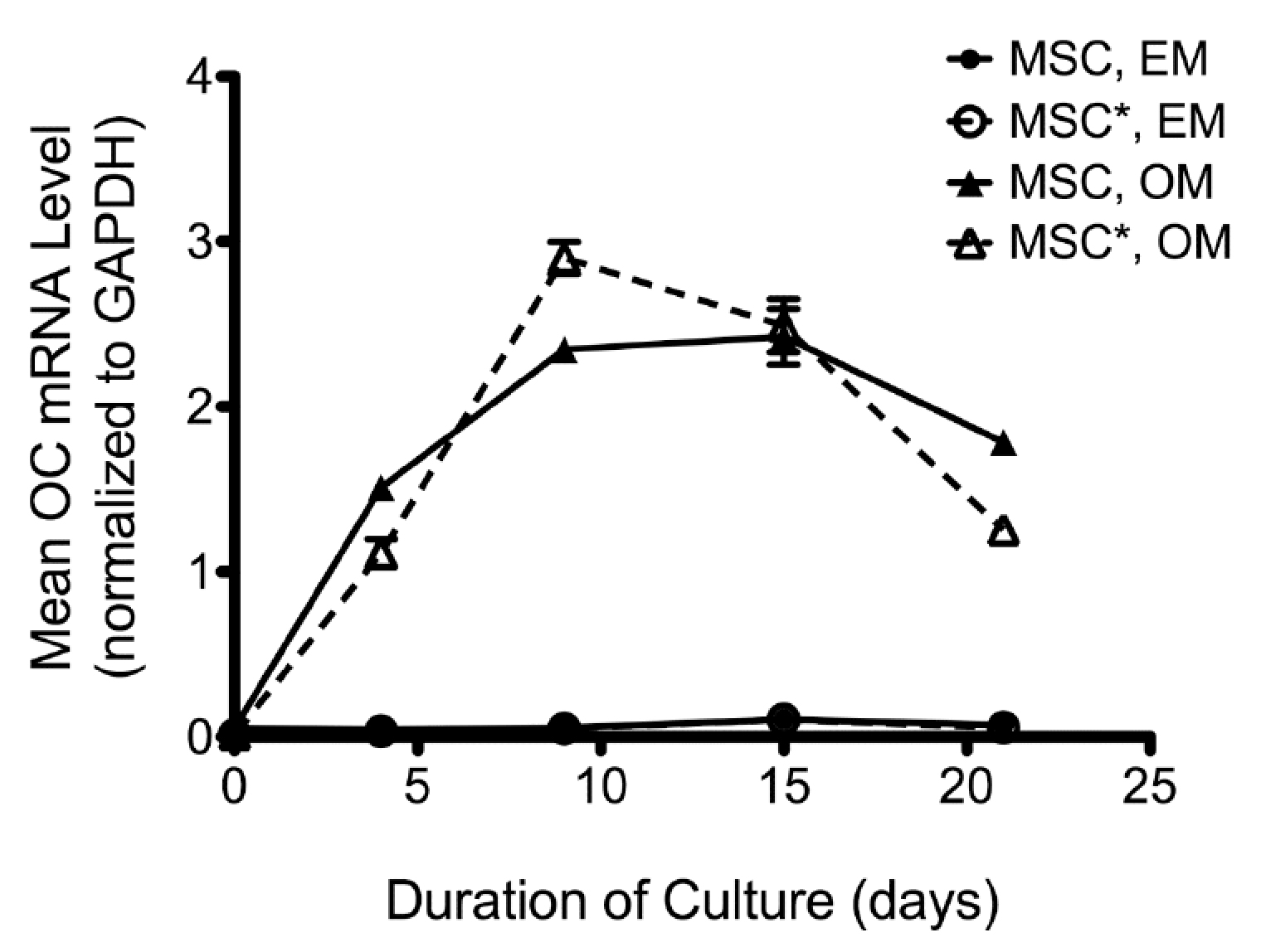

Supplement: Additional file 2: Figure S1. — DiI labeling does not affect MSC osteogenic gene expression. MSCs were labeled with DiI and cultured in EM or OM for 21 days. Osteocalcin (OC) mRNA expression was measured by real-time RT-PCR and normalized to GAPDH. Unlabeled MSCs were used as controls. Values shown are mean ± SD from cells isolated from a 61-year-old male donor, plated at 3,000/cm2. (JPEG 305 kb) [file 13287_2015_223_MOESM2_ESM.jpeg]

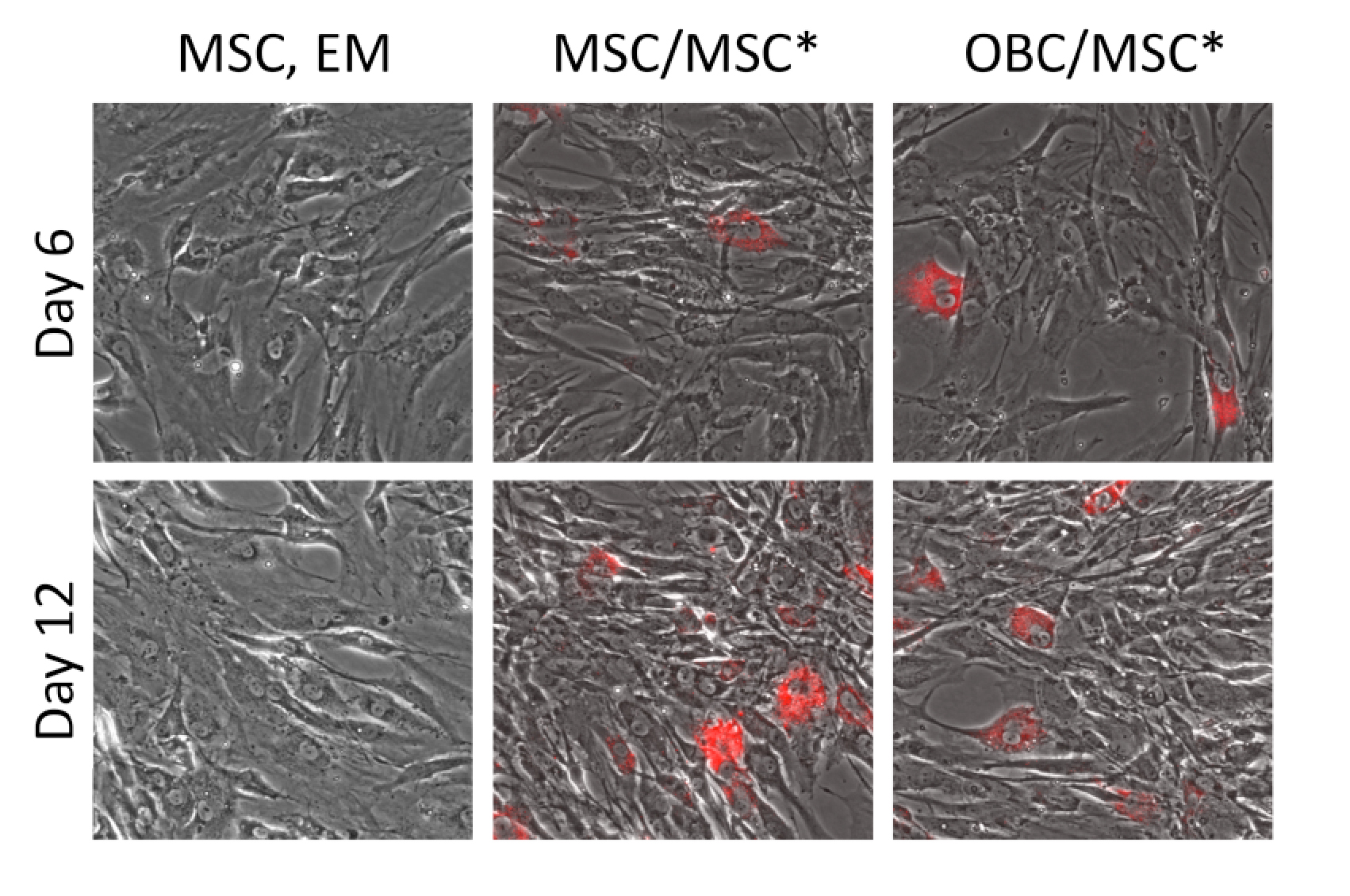

Supplement: Additional file 3: Figure S2. — Morphology of DiI-labeled MSCs in co-culture with unlabeled OBCs. Images of MSCs on culture days 6 and 12 show no obvious morphological differences due to co-culture with OBCs. Shown are unlabeled MSCs in EM; DiI-labeled MSCs mixed with unlabeled MSCs in OM as a control (MSC/MSC*); and DiI-labeled MSCs mixed with unlabeled OBCs in OM (OBC/MSC*). MSCs were derived from a 73-year-old male donor, plated at 9,000 cells/cm2 and cultured in a 1:4 ratio with OBCs induced from the same MSCs for 15 days prior to co-culture. MSC* indicates MSCs labeled with DiI (red). 10× magnification. (JPEG 1025 kb) [file 13287_2015_223_MOESM3_ESM.jpeg]

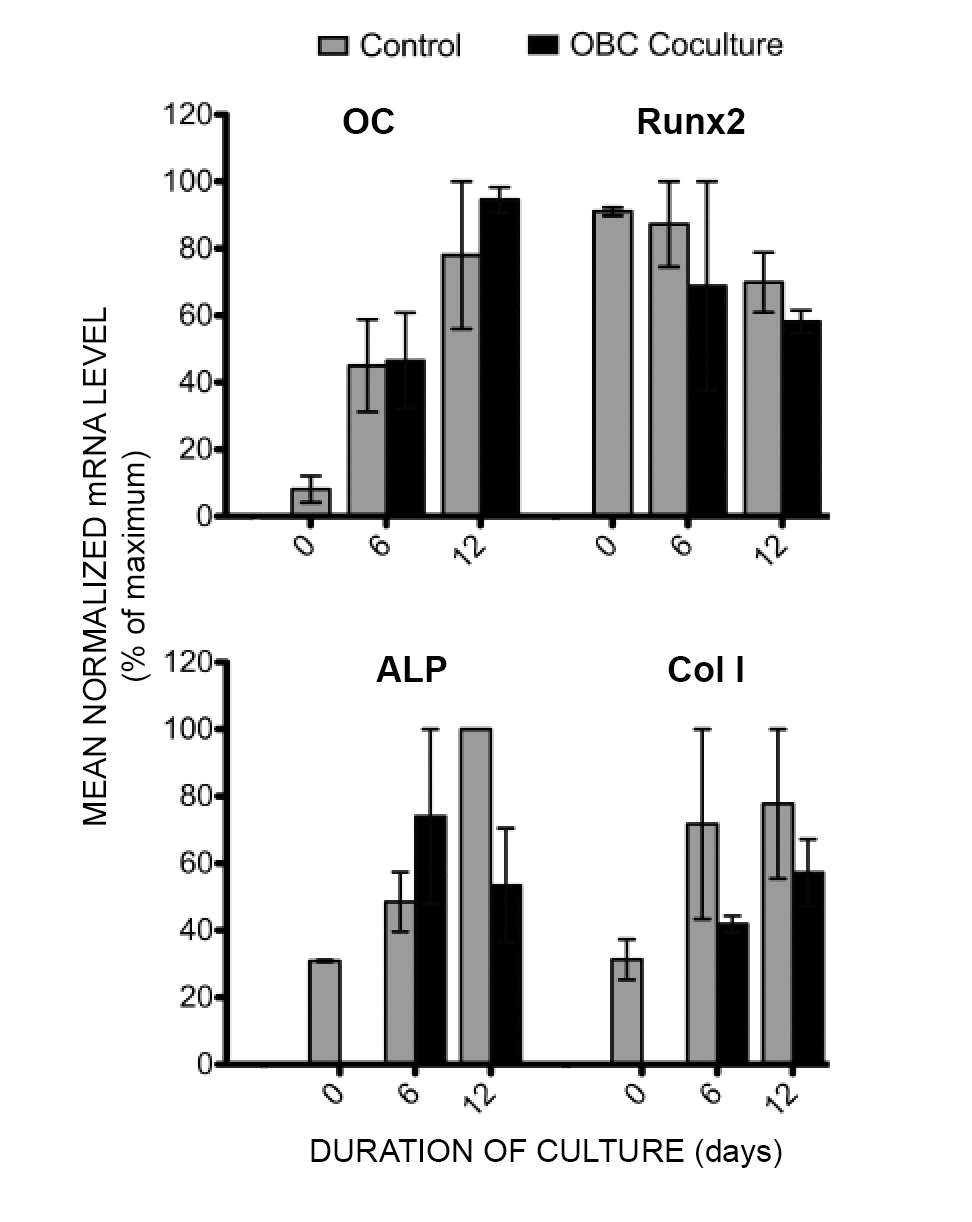

Supplement: Additional file 4: Figure S3. — Effects of OBC co-culture on MSC osteogenic gene expression is variable. mRNA levels of osteogenic genes (OC, Runx2, ALP, and Col I) in MSC control cultures and MSC/OBC co-cultures were measured via real-time RT-PCR, and normalized to GAPDH. Each value was then expressed as a percentage of the maximum level reached for that gene in cells from a given donor within a given experiment. Values shown are the mean ± SEM from two experiments. No statistically significant differences were observed. (JPEG 238 kb) [file 13287_2015_223_MOESM4_ESM.jpeg]

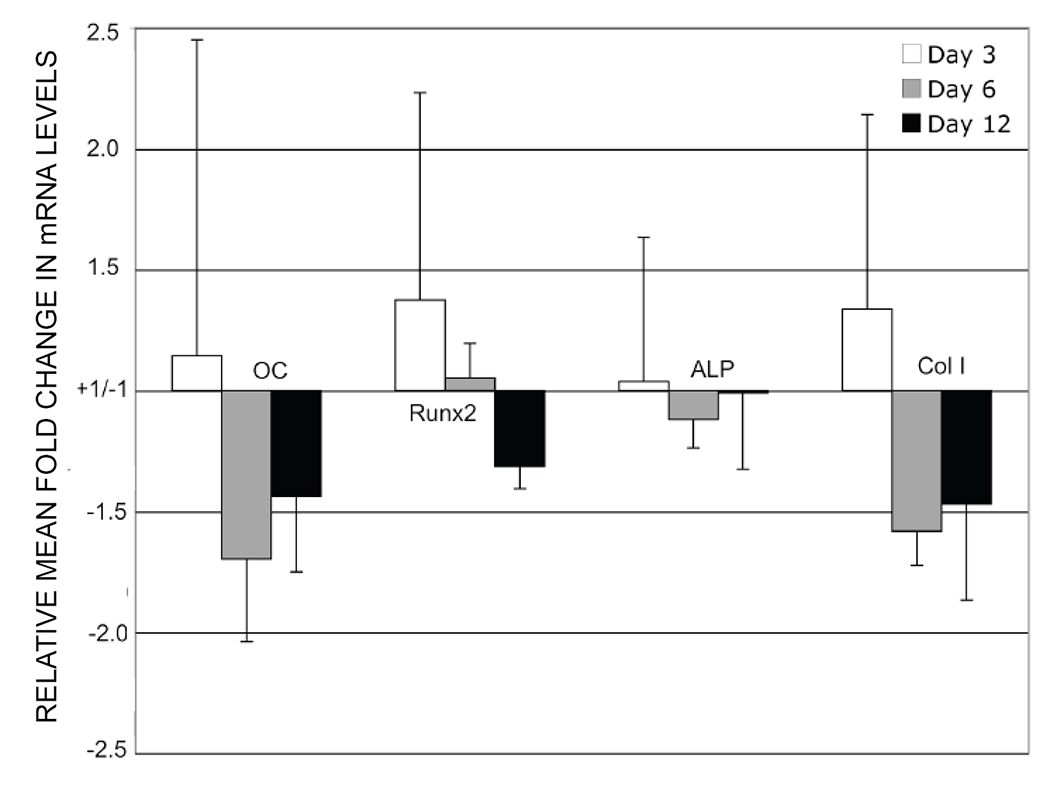

Supplement: Additional file 5: Figure S4. — Minimal effects of OBC-conditioned medium on MSC osteogenic gene expression. mRNA levels of OC, Runx2, ALP, and Col I measured via real-time RT-PCR (normalized to GAPDH) were compared between MSCs in OBC-conditioned OM (CM) versus those in the control aged OM (AOM) cultured for 3, 6 and 12 days. Mean fold changes on each day from three experiments are shown (mean ± SD). None of the changes in gene expression upon exposure to OBC-CM were greater than 1.7-fold, and none of them were statistically significant. (JPEG 147 kb) [file 13287_2015_223_MOESM5_ESM.jpeg]

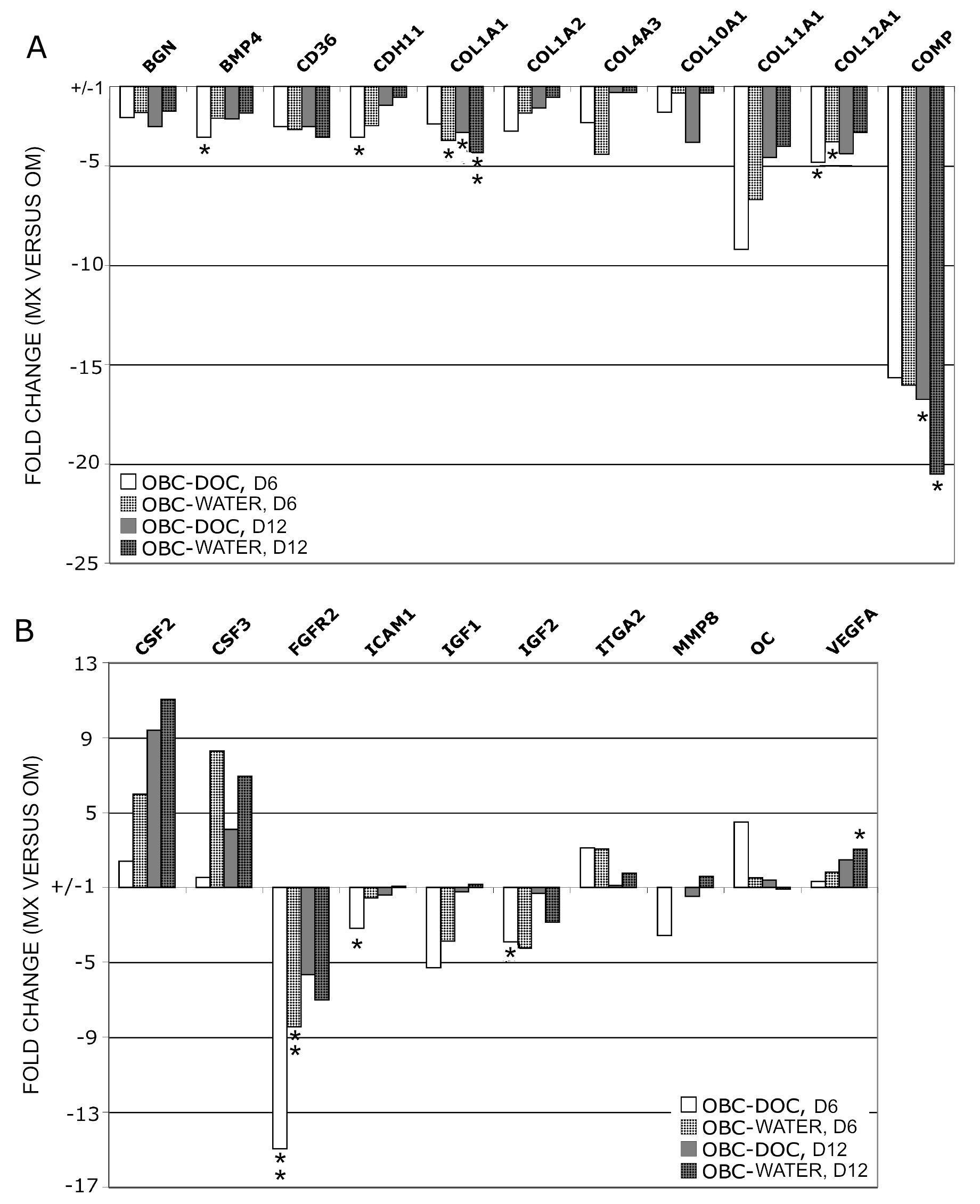

Supplement: Additional file 6: Figure S5 — Microarray analysis confirms the suppression of osteogenesis by OBC matrices. MSCs from three donors were cultured in OM on OBC matrices prepared by water or DOC lysis, and gene expression analyzed by real-time RT-PCR using the SuperArray System. Gene-of –interest CT values were normalized to the average of five housekeeping genes. Shown are those genes that showed mean fold changes > 3-fold when comparing MSCs on matrix to MSCs on plastic in OM at days 6 and 12. Genes were assigned to graph (A) or (B) based on alphabetical order. Asterisks indicate significance between an experimental condition and the OM plastic control. There was no statistical significance in any differences >3-fold between matrix treatments. *, p < 0.05; **, p < 0.01. (JPEG 427 kb) [file 13287_2015_223_MOESM6_ESM.jpeg]

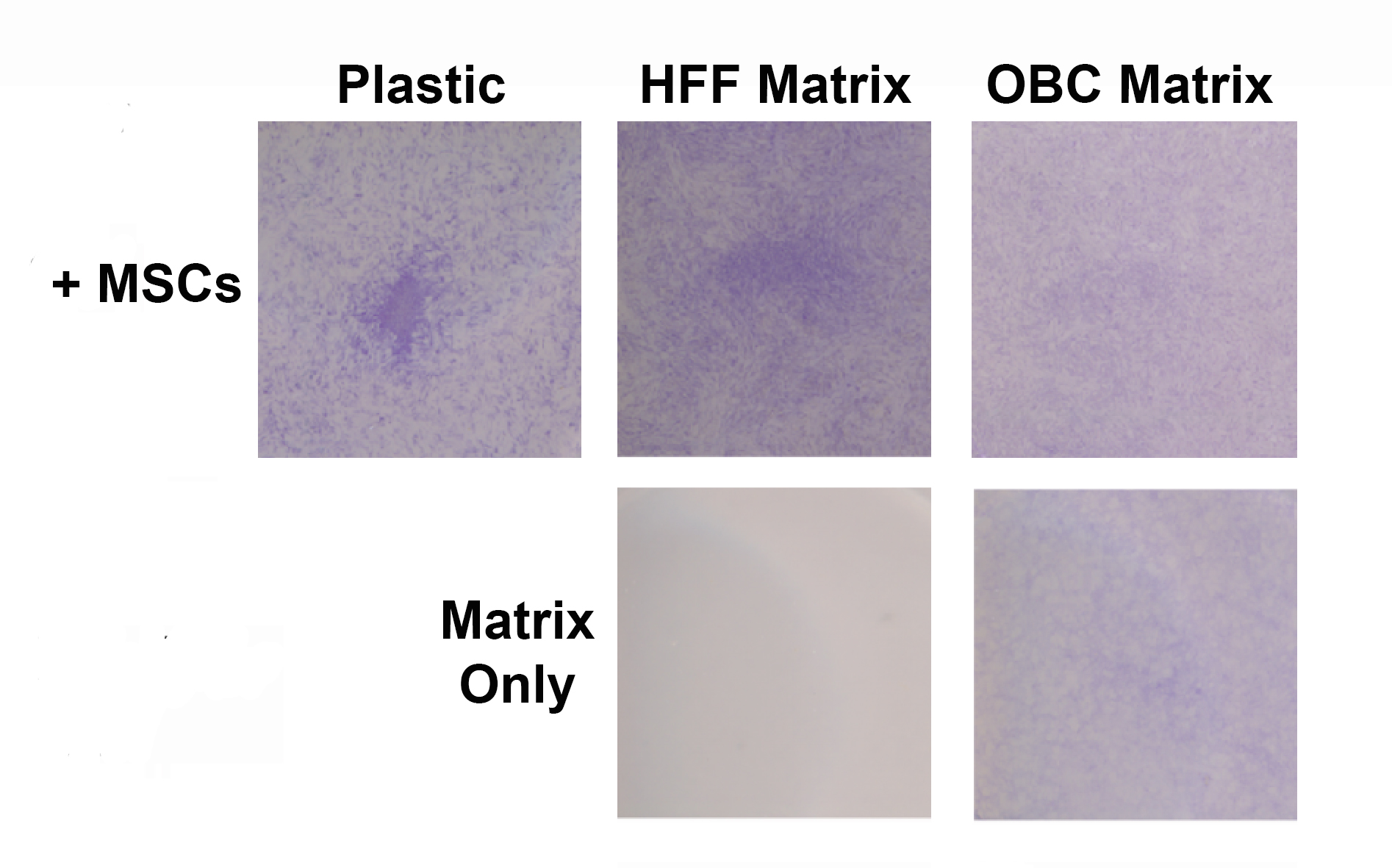

Supplement: Additional file 7: Figure S6. — Comparison of the matrix effect of human foreskin fibroblasts and osteoblastic cells on osteogenic differentiation of MSCs. HFF and OBC cultures were water-lysed and the resultant matrices used as a substrate for MSCs cultures maintained in osteogenic medium. ALP activity was detected histochemically. Non-MSC seeded HFF and OBC matrices were used as control. The results showed that HFF matrix did not suppress MSC osteogenesis, compared to OBC matrix. OBCs were derived from day-15 osteogenic culture of bone marrow derived MSCs from a 47-year-old female donor. (JPEG 637 kb) [file 13287_2015_223_MOESM7_ESM.jpeg]
