# Supplementary material for: Nascent osteoblast matrix inhibits osteogenesis of human mesenchymal stem cells in vitro
Source: Stem Cell Res Ther. 2015 Dec 22;6:258. doi: 10.1186/s13287-015-0223-x (PMC4688995; doi:10.1186/s13287-015-0223-x)
Supplement: Additional file 8: Table S2. — Protein symbols used for proteomic analysis. (DOCX 122 kb) [file 13287_2015_223_MOESM8_ESM.docx]

**Table S2: Protein Symbols Used for Proteomic Analysis**

| **Symbol** | **Entrez Gene Name** | **Location** |
| --- | --- | --- |
| ADAMTS4 | ADAM metallopeptidase with thrombospondin type 1 motif, 4 | EX |
| ANGPTL1 | Angiopoietin-like 1 | EX |
| ANXA1 | Annexin A1 | PM |
| ANXA2 | Annexin A2 | PM |
| ANXA6 | Annexin A6 | PM |
| COL6A1 | Collagen, type VI, alpha 1 | EX |
| COL6A2 | Collagen, type VI, alpha 2 | EX |
| COL6A3 | Collagen, type VI, alpha 3 | EX |
| COL7A1 | Collagen, type VII, alpha 1 | EX |
| EFEMP2 | EGF-containing fibulin-like extracellular matrix protein 2 | EX |
| EMILIN1 | Elastin microfibril interfacer 1 | EX |
| ERBB2 | v-erb-b2 erythroblastic leukemia viral oncogene homolog 2 | PM |
| ERK | Extracellular signal-regulated kinases | UN |
| FERMT2 | Fermitin family homolog 2 (Drosophila) | CYT |
| FLOT1 | Flotilin 1 | PM |
| FN1 | Fibronectin 1 | PM |
| GCNT1 | Glucosaminyl (N-acetyl) transferase 1, core 2 | CYT |
| GFRA2 | GDNF family receptor alpha 2 | PM |
| GNB2 | Guanin nucleotide binding protein (G protein), beta 2 | PM |
| HTRA1 | HtrA serine peptidase 1 | EX |
| ITGAE | Integrin, alpha E (antigen CD103) | PM |
| ITGB6 | Integrin, beta 6 | PM |
| ITGB8 | Integrin, beta 8 | PM |
| LRG1 | Leucine-rich alpha-2-glycoprotein 1 | EX |
| LTBP2 | Latent transforming growth factor beta binding protein 2 | EX |
| MGP | Matrix Gla protein | EX |
| PARVA | Parvin, alpha | CYT |
| RPL17 | Ribosomal protein L17 | CYT |
| RUNX2 | Runt-related transcription factor 2 | NU |
| SLC2A4 | Solute carrier family 2 (facilitated glucose transporter), 4 | PM |
| SNAI2 | Snail homolog 2 (Drosophila) | NU |
| TGFB1 | Transforming growth factor, beta 1 | EX |
| TGFBI | Transforming growth factor, beta-induced, 68kDa | EX |
| TP53 | Tumor protein p53 | NU |
| TPSB2 | Tryptase beta 2 | EX |

EX=extracellular space, CYT=cytoplasm, PM=plasma membrane, NU=nucleus, UN=unknown
